# Association between Catechol-O-methyltransferase rs4680 (G > A) polymorphism and lung cancer risk

**DOI:** 10.1186/s13000-014-0192-x

**Published:** 2014-10-04

**Authors:** Xiang Tan, Mingwu Chen

**Affiliations:** Department of Cardiothoracic Surgery, First Affiliated Hospital, Guangxi Medical University, Nanning, Guangxi China

**Keywords:** COMT, Polymorphism, Lung cancer, Meta-analysis

## Abstract

**Background:**

The association between the Val158Met polymorphism in the *catechol-O-methyltransferase* (*COMT*) gene and lung cancer risk remains controversial and inconclusive. Therefore, the meta-analysis was performed to provide a quality reevaluation of the association between the *COMT* Val158Met polymorphism and the risk of lung cancer.

**Methods:**

Two major public databases (Pubmed and Embase) and several Chinese databases were searched for eligible studies. Pooled odds ratios (OR) and 95% confidence intervals (CI) were calculated to estimate the strength of the association.

**Results:**

Five publications, including six individual studies with a total of 4,043 subjects (1,796 cases and 2,247 controls) regarding the association of *COMT* Val158Met polymorphism with lung cancer susceptibility were included in this meta-analysis. Overall, pooled analysis indicated that there was no significant association between *COMT* Val158Met polymorphism and lung cancer susceptibility under all genetic models. Likewise, no association was observed in the stratified analysis by ethnicity and control source, either. However, Val158Met polymorphism was shown to increase lung cancer risk among women (AG vs. GG, OR = 1.190, 95% CI = 1.001–1.422, *p* = 0.049).

**Conclusion:**

These findings suggested that the *COMT* l58Val/Met polymorphism confer genetic susceptibility to lung cancer among women. However, no evidence was found for the association with lung cancer risk in ethnicity and smoking status.

**Virtual slides:**

The virtual slide(s) for this article can be found here: http://www.diagnosticpathology.diagnomx.eu/vs/13000_2014_192

## Background

Lung cancer is the most frequently diagnosed cancer and is also considered as one of the most commonly lethal malignancies worldwide [1]. Despite the high morbidity and mortality of lung cancer, its etiology remains largely unknown. There are multiple factors and multistep processes contributing to lung cancer, among which environmental exposures, such as arsenic, asbestos, radon, and polycyclic aromatic hydrocarbons, specially tobacco smoking, are the major risk factor [2]. However, only a small proportion individual exposed to these carcinogens eventually develop lung cancer in the lifetime. Besides environmental risk, it has been believed that genetic factors also play a role in the development of lung cancer.

The *catechol-O-methyl transferase* (*COMT*) gene is located on the chromosome 22q11.2. The Val158Met (rs4680, G > A) single nucleotide polymorphism (SNP) in *COMT* gene have been identified in a protein coding region [3], which leads to a common substitution of methionine (Met) for valine (Val) at codon 158, This SNP has been reported to affect the activity of the COMT enzyme. For instance, individuals with the variant Met/Met genotype have a 3- to 4- fold lower COMT enzyme activity than those with wild-type Val/Val genotype [4]. COMT enzyme is involved in methylation of catechol estrogens, especially catechol estrogens formed from the 2- and 4-hydroxylations of estradiol. Reduced the activity of this enzyme may lead to increasing the accumulation of catecholestrogens, which may cause oxidative DNA damage through participating in redox cycling processes or quinones metabolism [4-7]. The DNA adducts can generate apurinic sites that may lead to cancer-initiating mutations [8] cause cellular transformation [9,10], and thereby initiate cancer development.

Up to date, a number of molecular epidemiologic case–control studies have shown the potential role of *COMT* Val158Met polymorphism in the risk of lung cancer. However, the results remain inconclusive and controversial. For example, Zhang et al. [7] had shown that *COMT* Val158Met was significantly associated with a reduced risk of NSCLC, whereas Lim et al. [11] had suggested that the variant A allele of *COMT* Val158Met polymorphism was positively associated with lung cancer in never-smokers. So far, no meta-analysis had been conducted to investigate the association between *COMT* Val158Met polymorphism and Lung cancer. With this in mind, we performed this meta-analysis with large samples to reevaluate this association.

## Methods

### Searching strategy and selection criteria

A comprehensive literature search was conducted in two comprehensive literature search engine (Pubmed, Embase), and several Chinese database, such as, Chinese Biomedical database, China National Knowledge Infrastructure (CNKI) and Wan fang (WF) Database until October 20, 2013. The search terms were “*catechol-O-methyltransferase* or *COMT*”, “Val158Met or rs4680 or G472A”, “polymorphism or variant or variation”, and “lung cancer or lung carcinoma”. Additionally, we identified extra studies by manually searching of the references lists of retrieved studies. All eligible studies included in our meta-analysis were conducted on human subjects, and published in either English or Chinese. If several studies overlapped with each, we only selected the one with the largest sample size or the one published most recently. The included studies have to meet the following criteria: (1) full-text studies were included, (2) designed as nested case–control, case–control, or cohort study, (3) estimated the association between *COMT* Val158Met variant and lung cancer, (4) odds ratio (OR) and the corresponding 95% confidence interval (CI) were provided, and (5) provided the sufficient genotypic or allelic information to estimate.

### Data extraction

Information was extracted carefully from all eligible studies independently by two investigators (X. Tan and MW. Chen). All disagreements were resolved by fully discussing between two investigators until a consensus was reached. The following data were collected from each study: first author’s surname, publication date, country, ethnicity, source of control, genotyping method, and genotype counts (GG, GA, and AA) of cases and controls (Table 1).

**Table 1:** **Studies and data included in this meta-analysis**

**Investigator**	**Year**	**Race**	**Country**	**Case**			**Control**			**HWE** ^**a**^	**Control source**	**Methods**	**NOS** ^**b**^ **score**
				**AA**	**AG**	**GG**	**AA**	**AG**	**GG**	***p*** **a**			
Zhang et al. [7]	2013	Asian	China	11	69	120	19	78	103	0.454	HB^c^	Sequence	8
Lim et al. [11]	2012	Asian	Singapore	39	220	284	63	353	549	0.539	HB	PCR^e^	7
Cote et al. [18]	2009	Caucasian	USA	102	205	78	114	197	92	0.696	PB^d^	PCR	8
Cote et al. [18]	2009	Black	USA	10	46	56	14	47	59	0.332	PB	TaqMan	8
Zienolddiny et al. [20]	2008	Caucasian	Norway	32	62	163	8	60	202	0.182	PB	Sequence	8
Gemignani et al. [19]	2007	Caucasian	European	59	144	83	75	146	81	0.569	HB	PCR	7

*p*a for Hardy–Weinberg equilibrium in control group; ^b^for Newcastle-Ottawa Scale. ^c^for hospital-based; ^d^for population-based; ^e^for polymerase chain reaction.

### Quality assessment

The Newcastle-Ottawa Scale (NOS) was used to assess the methodological quality of studies included in our meta-analysis. The NOS consists of eight items that are further classified into three categories: selection, comparability, and outcome. A star system was created to Semi-quantitatively weigh up the study quality. The comparability group was granted two stars whereas other each items awarded a maximum of one star in highest quality studies. The range of 1–9 stars in the NOS was adopted for estimation, with more stars indicating a higher quality study [12]. Since standard criteria had not been established, we considered study that was rated seven or more stars as a high quality study in the current meta-analysis.

### Statistical analysis

The strength of association between *COMT* Val158Met (G > A) polymorphism and lung cancer risk was assessed by the pooled ORs with 95% CIs. The pooled estimates were performed under several genetic models, including allele comparison model (A vs. G), homozygote comparison (AA vs. GG), heterozygote comparison (AG vs. GG), dominant model (AA/AG vs. GG), and recessive model (AA vs. AG/GG).

Chi-square-based Q test and *p*-value were used to test the statistical heterogeneity between studies. A *P* >0.10 and *I*^2^ < 25% for the Q test indicated that no heterogeneity existed among studies. And 25% < *I*^2^ < 50% indicated that there was moderate heterogeneity. If there was no heterogeneity, the fixed-effects model was adopted in Mantel–Haenszel method; if *I*^2^ > 50% or *p*-value < 0.1, the random-effects model was performed using the DerSimonian and Laird method if *I*^2^ > 50% or *p*-value < 0.1 [13].

Hardy–Weinberg equilibrium (HWE) was checked in controls by the chi-square test, and a *p*-value less than 0.05 represented significant deviation from HWE [14]. An asymmetric plot demonstrated a potential publication bias. The Begg’s and Egger’s test were carried out to detect potential publication bias, with a *P* < 0.05 indicating that there was a significant publication bias in the study [15].

The false-positive report probability (FPRP) [16,17] was carried out to validate the significant results. A FPRP value of 0.2 threshold and a prior probability of 0.01 were preset to detect an OR of 1.50 (for risk effects) or 0.67 (for protective effects) for an association between the studied SNP and lung cancer risk. Only significant associations with a FPRP value less than 0.2 were considered as noteworthy.

Moreover, we performed subgroup analyses by the ethnic subgroup (Asian and Caucasian), control source (Population-based and Hospital-based) and smoking status (Never-smokers and Ever-smokers). All statistic except for FPRP analysis, were performed by using the Stata software version11.1 (Stata Corporation. USA). And FPRP was conducted by using SAS software (version 9.1; SAS Institute, Cary, NC). All *P* values were two-sided, and a *P* value less than 0.05 were considered statistically significant.

## Results

### Characteristics of relevant studies

**Figure 1:**
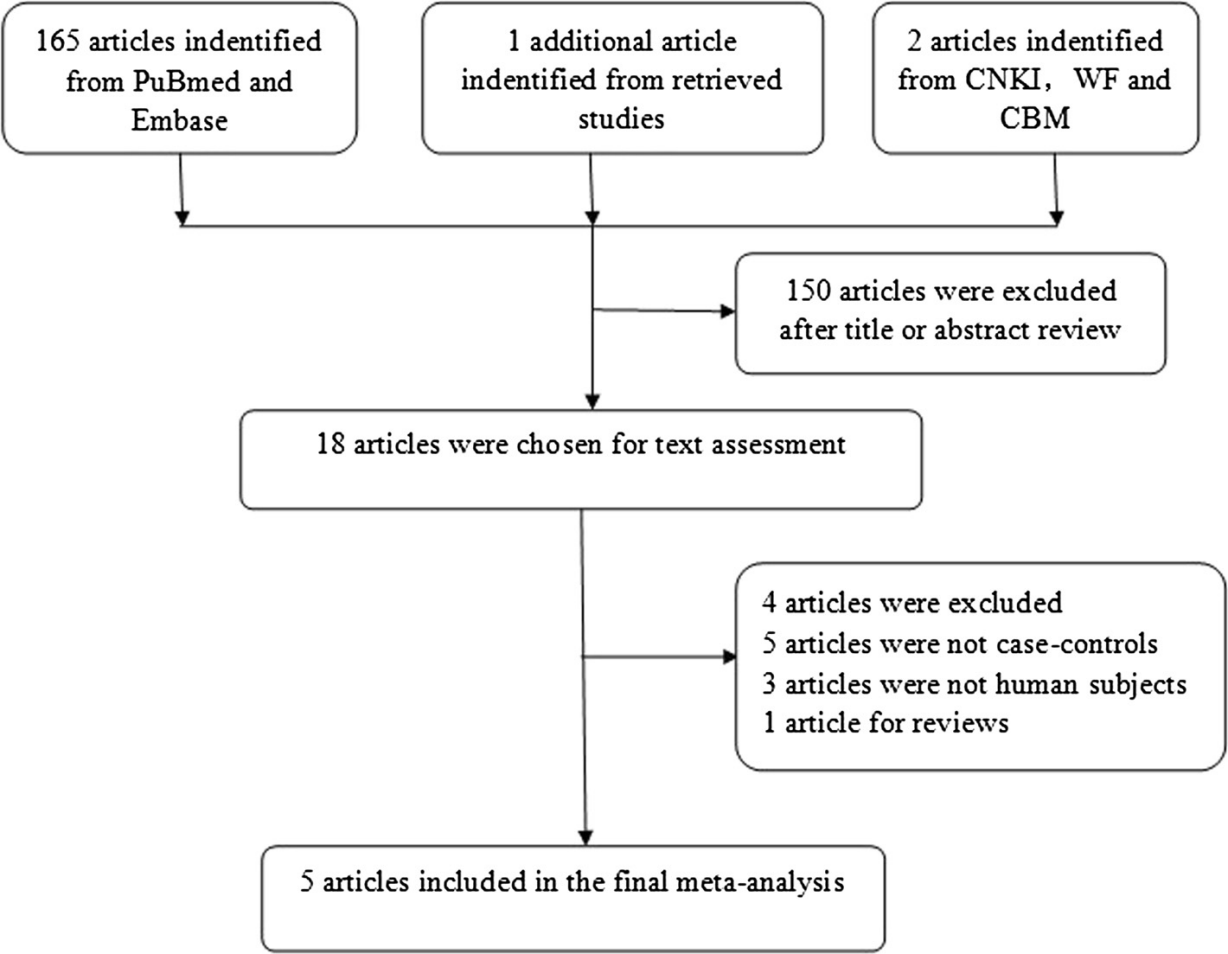
**The publication selection process of this meta-analysis.**

According to the previously described search strategy and inclusion criteria, the literature selection process was shown in Figure 1. Among all the eligible studies, study by Cote et al. [18] reported the genotype frequency of both Africans and Caucasians; thus the data were collected separately based on ethnicities and considered as two studies. Therefore, five publications [7,11,18-20], consisting of six individual studies with a total of 4,043 subjects (1,796 cases, 2,247 controls) regarding association of *COMT* Val158Met polymorphism with lung cancer risk were included in this meta-analysis. The study characteristics are summarized in Table 1. In term of the ethnicity, there were 2 studies [7,11] conducted among Asians, 3 studies [18-20] among Caucasians, and the remaining 1studies [18] among Africans. Regarding the source of control, there were 3 population-based studies and 3 hospital-based studies. Smoking is a major factor contributing to the development of lung cancer. Only 3 studies [7,11,20] provided genotype counts of cases and controls separately by the smoking status and separated the raw data of genotype was separated for further analysis. In addition, 2 publications [11,18], including 3 individual studies performed among women were included in the meta-analysis.

### Meta-analysis results

**Figure 2:**
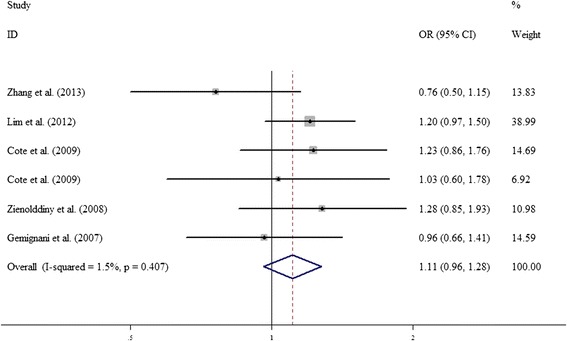
**Forest plots of COMT Val158Met polymorphism and lung cancer in the overall analysis using the fixed-effect dominant model (AG vs. GG).**

The meta-analysis results on the association between *COMT* Val158Met polymorphism and the risk of lung cancer are shown in Table 2. When between-study heterogeneity was significant, the random-effects model was performed in our analysis; otherwise, fixed-effects model was adopted used. By pooling eligible studies together, the results derived from random-effect models indicated that variant allele (A) of *COMT* Val158Met was not associated with lung cancer (A vs. G, OR = 1.052, 95% CI = 0.837–1.322, *p* = 0.664). Similarly, no significant associations were observed under all other genetic models (homogenous: AA vs. GG, OR = 1.088,95% CI = 0.677–1.749, *p* = 0.729; heterogeneous: AG vs. GG, OR = 1.107, 95% CI = 0.962–1.275, *p* = 0.157; dominant: AA/AG vs. GG, OR = 1.085, 95% CI = 0.867–1.357, *p* = 0.477; recessive: AA vs. AG/GG, OR = 1.037, 95% CI = 0.686–1.568, *p* = 0.863) (Table 2, Figure 2).

**Table 2:** **Stratified analyses of the COMT gene polymorphism and lung cancer risk**

**Variable**	**n**	**AA vs. GG**			**AG vs. GG**			**AA/AG vs. GG**			**AA vs. AG/GG**		
		**OR (95% CI)**	***p*** ^**a**^	***p*** ^**b**^	**OR (95% CI)**	***p*** ^**a**^	***p*** ^**b**^	**OR (95% CI)**	***p*** ^**a**^	***p*** ^**b**^	**OR (95% CI)**	***p*** ^**a**^	***p*** ^**b**^
Total	6	1.088 (0.677–1.749)	0.729	0.001	1.107 (0.962–1.275)	0.157	0.407	1.085 (0.867–1.357)	0.477	0.03	1.037 (0.686–1.568)	0.863	0.002
Ethnicity													
Asian	2	0.823 (0.351–1.930)	0.654	0.054	0.991 (0.634–1.549)	0.968	0.055	0.949 (0.566–1.592)	0.842	0.02	0.848 (0.438–1.642)	0.626	0.121
Caucasian	3	1.480 (0.627–3.491)	0.371	0.001	0.993 (0.797–1.238)	0.227	0.544	1.210 (0.845–1.732)	0.299	0.049	1.350 (0.642–2.839)	0.429	0.001
Control source													
Hospital-based	3	0.836 (0.531–1.315)	0.438	0.112	1.061 (0.892–1.262)	0.505	0.136	0.946 (0.687–1.304)	0.735	0.047	0.854 (0.612–1.192)	0.354	0.236
Population-based	3	1.560 (0.562–4.336)	0.393	0.001	1.204 (0.945–1.534)	0.134	0.816	1.277 (0.929–1.755)	0.132	0.153	1.434 (0.519–3.966)	0.487	0.001
Smoking status													
Never-smokers	2	0.846 (0.330–2.169)	0.728	0.043	1.064 (0.574–1.972)	0.844	0.012	1.016 (0.518–1.992)	0.963	0.004	0.842 (0.427–1.658)	0.618	0.13
Ever-smokers	2	2.326 (0.503–10.756)	0.28	0.014	0.994 (0.590–1.677)	0.983	0.096	1.174 (0.548–2.517)	0.680	0.010	2.394 (0.619–9.262)	0.206	0.028

^a^the pooled p value; ^b^ *p* value for heterogeneity test.

**Figure 3:**
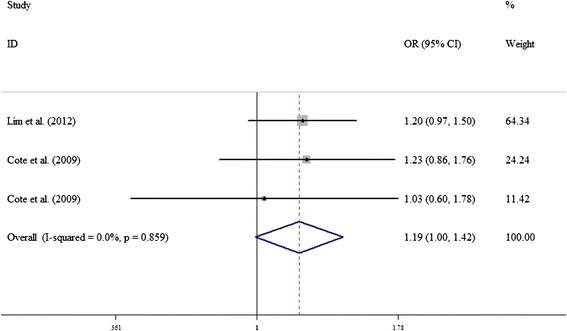
**Forest plots of COMT Val158Met polymorphism and lung cancer among women using the fixed-effect model (AG vs. GG).**

Moreover, in the stratified analysis by ethnicity, there was no significant association between *COMT* Val158Met polymorphism and lung cancer risk among Caucasians and Asians (Table 2). Further stratified analysis by source of controls or smoking status observed, no significant association (Table 2). However, we found a borderline significant association between risk Vall58Met polymorphism and the risk of lung cancer among women under the heterogeneous model (AG vs. GG, OR = 1.190, 95% CI = 1.001–1.422, *p* = 0.049) (Figure 3).

FPRP analysis indicated that, for a prior probability of 0.1, assuming that the OR for specific genotype was 0.67/1.50 (protection/risk), Val/Met carriers for women were not increased lung cancer risk at the prior probability level of 0.1or 0.01 (power = 0.999, f0.1 = 0.306, f0.01 = 0.829). However, these finding need further validation with larger sample sizes, because of some possible bias that resulted from small sample size in subgroups.

### Test for heterogeneity, sensitive analysis, and test for publication bias

Significant heterogeneity was observed in the overall pooled analysis, whereas, the degree of heterogeneity was decrease in the stratified analysis. In order to assess the stability of the present meta-analysis, sensitivity analysis was performed by deleting one study at each time (data not shown) and recalculating ORs and 95% CIs. Overall, the pooled estimates were not materially altered upon the removal of any of single study, suggesting that the stability of the results.

**Figure 4:**
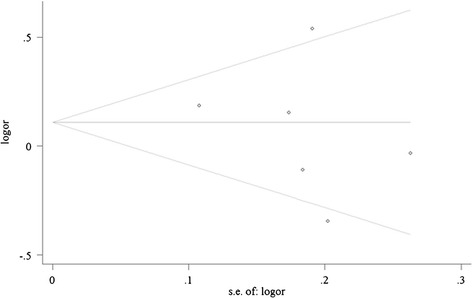
**Begg’s funnel plot of the meta-analysis of lung cancer risk and COMT Val158Met polymorphism (AA + AG vs. GG).** Begg's funnel plot with pseudo 95% confidence limits.

Begg’s funnel plot and Egger’s test were conducted to estimate the publication bias. The shape of the funnel plot revealed no evidence of obvious asymmetry in this meta-analysis (Figure 4). Likewise, Begg’s and Egger’s test indicated no evidence of publication bias, either (AA versus GG, Begg’s test, *P* = 0.851, Egger’s test, *p* = 0.810; AG versus GG, Begg’s test, *P* = 0.188, Egger’s test, *p* = 0.345; dominant model, Begg’s test, *P* = 0.348, Egger’s test, *p* = 0.529; recessive model, Begg’s test, *P* = 0.851, Egger’s test, *p* = 0.599).

## Discussion

Lung cancer is the leading cause of cancer-related mortality worldwide. Lung carcinogenesis is a complicated long multi stage process, involving the interplay of cancer susceptibility genes and specific environmental exposures [21]. For example, a personal history of cigarette smoking probably is the most important contributor to the imitation and development of tobacco-related lung cancer. Apart from smoke exposure, genome-wide association studies (GWAS) has provided a strong evidence that tobacco-dependent lung cancer patients had a susceptibility region in chromosome 15q25.1, including some genes (CHRNB4, CHRNA3, and CHRNA5) that encode for nicotinic acetylcholine receptors [22]. These results suggest that genetic and environmental risk factors may collaboratively contribute lung cancer carcinogenesis.

Numerous studies have adopted a candidate gene approach to explore the effects of genetic variations on the genetic susceptibility to lung cancer. A large proportion of relevant studies has been focused on SNPs in the genes coding for enzymes involved in tobacco carcinogen metabolism (e.g. CYP1A1 and NQO1) and genes involved in DNA repair (e.g. XRCC1) [23,24]. Moreover, so many meta-analysis had investigated the association between the gene polymorphisms and the risk of lung cancer. The meta-analysis of Tan et al. [25] had reported a correlation between (ERCC2) Lys751Gln polymorphism and an increased risk of lung cancer based on 23,370 subjects. Wang et al. [26] performed a meta-analysis showed that there was no association between IL-6 rs1800796 polymorphism and lung cancer, etc. Therefore, Meta-analysis can provide a quantitative summary of the available data that will be stronger evidence for identifying the association between gene variations and cancer risk.

*COMT* polymorphism has also gained more and more attentions in this field. In addition, COMT is a rate-limiting enzyme that played a role in the detoxification of catechol estrogens. Reduced activity of this enzyme might cause the accumulation of catechol estrogens, which may sequentially lead to oxidative DNA damage [18,27]. Estrogen receptor β is expressed in lung tissue, and estrogen receptor antagonists could significantly inhibit growth in non-small-cell lung cancer cell lines [28,29]. There might be the oxidative metabolism of 17βestradiol and estrone to catechol estrogens that plays a key role in carcinogenesis [30].

Several epidemiologic studies have reported the role of *COMT* Val158Met polymorphism in the risk of lung cancer. Zienolddiny et al. [20] suggested that the exonic SNP (Val158Met) of the COMT gene was associated with an increased risk of lung cancer. Intriguingly, Zhang et al. [7] reported an opposite results in his research. Therefore, the previous results were conflicting and inconclusive. Although only limited studies were included, our meta-analysis provided a new evidence of the null association between *COMT* Val158Met polymorphism and overall lung cancer risk. To the best of our knowledge, this is the first meta-analysis that has evaluated the relationship between the *COMT* Vall58Met polymorphism and the risk of lung cancer.

Furthermore, *COMT* Vall58Met polymorphism appeared to have no influence on the risk of lung cancer either, when studies were stratified by ethnicity, source of controls and smoking status. There might be due to different genetic backgrounds or some other potentially suspected factors, which influenced our research.

The stratified analysis by sex observed a borderline significant association of *COMT* Val158Met polymorphism among women. To avoid false positive result from multiple comparison, we performed the FPRP analysis for these significant findings. Nevertheless, Val/Met carriers were not increased lung cancer risk at the prior probability level of 0.1or 0.01. There are several possible explanations for these findings. Some findings in the stratified analysis may be discovered by chance because of multiple comparisons and the limited sample size in the subgroups. Given only a few studies were included in this meta-analysis, it was hard to draw firm conclusions from this current analysis. Particularly, the borderline significant association for women was just based on 2 studies. Therefore, these findings should be interpreted with caution.

We used the methods of the Newcastle-Ottawa Scale (NOS) to assess the quality of the studies included in our meta-analysis. As a result, three studies [7,18,20] were rewarded eight stars, while two studies [11,19] were rewarded seven stars according to the NOS assessment system. Zhang et al. [7] and Lim et al. [11] had identified the association of *COMT* Val158Met polymorphism with the risk of lung cancer in Chinese non-smokers. The limitations of study by Zhang et al. [7] was that the sample size was small, and they did not analyze the effects of second-hand smoke and exposure to cooking smoke on the risk of NSCLC. The strengths of study by Lim et al. [11] included homogeneity with regard to gender and ethnicity and the large number of never-smokers. However, this study might be subjected to biases that exist in the hospital-based case–control studies. The individual data about environmental factors in the study by the Cote et al. [18] were relatively were detailed, including information on past medical and reproductive histories, occupational exposures, BMI, etc. Therefore, this study was able to explore the gene–gene and gene–environment interactions that influence an individual lung cancer risk. Nevertheless, the majority of the subjects in the study were lung adenocarcinomas. Thus, the findings might not be representative of lung cancer. Zienolddiny et al. [20] and Gemignani et al. [19] performed the genotyping of more than one hundred polymorphisms in genes involved in carcinogen and xenobiotic metabolism. However,the sample sizes of both studies were moderate. Therefore, there probably existed potential confounding false positives.

Although we took considerable efforts to collect all available data to investigate the association of *COMT* Vall58Met polymorphism and its correlation with lung cancer risk, there were also some limitations to be addressed. Firstly, there were only a few studies investigating relationship between *COMT* polymorphisms and lung cancer risk, therefore, the sample size of our meta-analysis was relatively small. Moreover, this meta-analysis might be also subjected from population stratification, a systematic difference in allele frequencies between subpopulations. In some cases, positive finding of an association study could result from population stratification, instead of a true association between studies SNP and disease. Secondly, our meta-analysis was based on unadjusted estimates, while a more precise analysis should be performed if individual data such as age, smoking status, lifestyle and environmental factors were available. These factors may be sources of the heterogeneity. Thirdly, only five studies were retrieved from Pubmed, Embase, China National Knowledge Infrastructure, and Medline data base. And unpublished studies and conference abstracts were excluded from the current study. Although Begg’s funnel plot, Begg’s test, and Egger’s test were conducted to estimate the publication bias. Lack of publication bias in this study might be due to the fact that Egger’s test was not very powerful with only a few studies in the meta-analyses, the results must be considered cautious. In spite of these limitations, our research was more powerful than each of single study.

## Conclusions

In conclusion, no significant association was found between *COMT* Vall58Met polymorphism and lung cancer risk in the overall analysis. However, a borderline significant association indicated that *COMT* Vall58Met polymorphism might increase lung cancer susceptibility. Finally, high-quality case–control studies with large sample sizes will be required to confirm these findings.
